# Symptom patterns and life with post-acute COVID-19 in children aged 8–17 years: a mixed-methods study protocol

**DOI:** 10.3399/BJGPO.2022.0149

**Published:** 2023-04-05

**Authors:** Alice Faux-Nightingale, Claire Burton, Helen Twohig, Milica Blagojevic-Bucknall, Will Carroll, Carolyn A Chew-Graham, Kate Dunn, Francis Gilchrist, Toby Helliwell, Oliver Lawton, Sarah Lawton, Christian Mallen, Benjamin Saunders, Danielle van der Windt, Victoria Welsh

**Affiliations:** 1 School of Medicine, Keele University, Keele, Newcastle, UK; 2 Staffordshire Children's Hospital at Royal Stoke, Stoke-on-Trent, United Kingdom

**Keywords:** COVID-19, child health, primary health care, long-COVID, symptom patterns

## Abstract

**Background:**

While there is a substantial body of knowledge about acute COVID-19, less is known about long-COVID, where symptoms continue beyond 4 weeks.

**Aim:**

To describe longer-term effects of COVID-19 infection in children and young people (CYP) and identify their needs in relation to long-COVID.

**Design & setting:**

This study comprises an observational prospective cohort study and a linked qualitative study, identifying participants aged 8–17 years in the West Midlands of England.

**Method:**

CYP will be invited to complete online questionnaires to monitor incidences and symptoms of COVID-19 over a 12-month period. CYP who have experienced long-term effects of COVID will be invited to interview, and those currently experiencing symptoms will be asked to document their experiences in a diary. Professionals who work with CYP will be invited to explore the impact of long-COVID on the wider experiences of CYP, in a focus group. Descriptive statistics will be used to describe the incidence and rates of resolution of symptoms, and comparisons will be made between exposed and non-exposed groups. Logistic regression models will be used to estimate associations between candidate predictors and the development of long-COVID, and linear regression will be used to estimate associations between candidate predictors. Qualitative data will be analysed thematically using the constant comparison method.

**Conclusion:**

This study will describe features and symptoms of long-COVID and explore the impact of long-COVID within the lives of CYP and their families, to provide better understanding of long-COVID and inform clinical practice.

## How this fits in

While there is increasing knowledge about acute COVID-19, little is known about long-COVID or how it affects CYP. Use of mixed methods will allow this study to develop a better understanding of the features and symptoms of long-COVID and how it affects the lives of CYP and their families. This knowledge can be used to inform future clinical practice.

## Introduction

Severe acute respiratory syndrome coronavirus 2 (SARS-CoV-2) infection, which causes COVID-19, usually results in mild illness in CYP, and few life-threatening complications are reported for this age group.^1^ However, evidence suggests that a small subset within this population may face longer-term consequences after an acute COVID-19 infection.^2^ Long-COVID describes cases where symptoms continue beyond the acute infection,^3,4^ although the exact definition varies in the literature.^5,6^ Within this study, long-COVID will be defined as physical and mental symptoms that last more than 4 weeks after an acute episode of COVID-19, describing both ongoing symptomatic (or post-acute) COVID-19 (5–12 weeks after onset) and post-COVID-19 syndrome (12 weeks or more).^7^

The most recent UK Office for National Statistics (ONS) estimates of self-reported long-COVID, using data from the Coronavirus (COVID-19) Infection Survey, suggest 2.0 million people are experiencing symptoms persisting for more than 4 weeks after acute COVID-19.^8^ Existing findings suggest that prevalence of long-COVID is low in CYP, although figures vary according to study, with heterogeneity within the sample sizes, study method, and period of time since the acute illness.^2,9–11^ Recent research has reported low-level evidence to suggest that vaccination before infection with SARS-CoV-2 may reduce risk of long-COVID.^12^

A wide range of symptoms of long-COVID have been identified in CYP, reflected in the authors' systematic review of the literature (reference: PROSPERO CRD42020226624), of which fatigue, headache, and sore throat have been commonly reported.^9^ It is important to consider not only the symptom presentation of long-COVID, but also the wider impact of the illness on the lives of CYP, including factors such as education and social development, which have already been affected by the COVID-19 pandemic.^13^

Research to identify the ongoing needs of CYP in relation to long-COVID is critical. The UK NHS *Long Term Plan* highlights that ‘*the needs of children are diverse, complex and need a higher profile*’^13^ and, while studies have investigated the wider impact of the COVID-19 pandemic on the mental health and wellbeing of CYP,^14–17^ it is important to understand how long-COVID affects CYP and their social interactions. Better understanding and definition of the clinical elements of long-COVID will inform clinical practice and help to develop beneficial treatment plans and interventions for those with the condition.

This study aims to investigate the longer-term effects of an acute COVID-19 infection in CYP aged 8–17 years, residing within the West Midlands, addressing the following objectives:

1. To describe the presentation, spectrum, and trajectory of symptoms lasting longer than 4 weeks after an acute episode of COVID-19 in CYP aged 8–17 years.
2. To determine the incidence of long-COVID following COVID-19 infection in a cohort of CYP.
3. To identify predictors of the development of long-COVID in CYP.
4. To describe the physical, psychological, and social outcomes in CYP with long-COVID compared with those without long-COVID.
5. For all CYP aged 8–17 years, to investigate the association of predictors, including having long-COVID and vaccination status, with general health-related quality of life (HRQoL) at 6 months post-study inception.
6. To explore the impact of long-COVID on the lives of CYP and their families (including personal, family, social, and educational effects).
7. To describe preferences for and understanding of: information and treatments; care needs and priorities; and important outcomes for CYP with long-COVID in primary care settings.

## Method

### Study design

**Figure 1.**
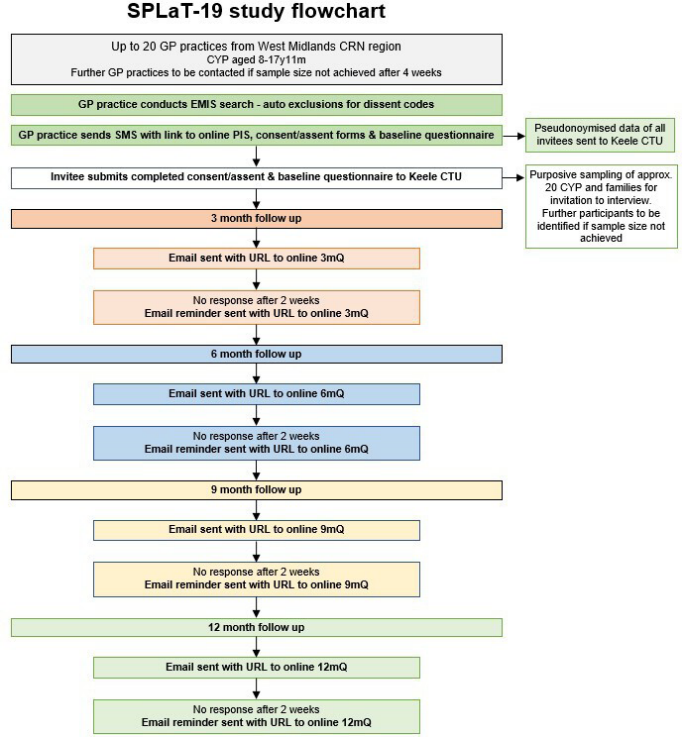
SPLaT-19 study flowchart. CRN = Clinical Research Network. CTU = clinical trials unit. CYP = children and young people. PIS = participant information sheet.

The Symptom Patterns and Life with Longer Term COVID-19 in children and young people (SPLaT-19) study comprises the following: (1) an observational prospective cohort study (SPLaT-C); and (2) a linked qualitative study (SPLaT-Q). For a flow chart of the study protocol see Figure 1.

### Patient and Public Involvement and Engagement (PPIE)

The SPLaT-19 study team includes a young lay co-investigator who has been involved with developing the study protocol, topic guides, and grant application. The West Midlands Young Persons' Steering Group (generationr.org.uk/birmingham) provided input to the initial development of the study and they will remain integrated into the project, providing input into the interpretation of the analysis and dissemination of the findings.

### Cohort study

#### Study setting

Up to 20 GP practices within the National Institute for Health and Care Research (NIHR) Clinical Research Network (CRN) West Midlands will be recruited to the study.

#### Participants

Participants will be identified via participating GP practices. All CYP aged 8–17 years registered at a participating practice and satisfying the inclusion and exclusion criteria will be invited to participate.

#### Inclusion criteria

All CYP aged between 8 and 17 years are eligible for this study, whether they have had acute COVID-19 or not. Prospective participants must have a mobile number in their GP record, parental or personal.

#### Exclusion criteria

CYP with dissent codes on medical records for research and/or messaging services will not be contacted.

#### Invitation and recruitment

CYP who meet the inclusion criteria will be sent an SMS inviting them to the study. The SMS will contain an introduction to the study with a URL link to the study homepage where there will be further information and the participant information sheets. CYP and parents or guardians who are willing to participate can assent or consent through e-consent forms on the website. Participants identified as experiencing longer-term effects of COVID-19 will be invited to the qualitative study. A summary of the patient identification, invitation, and recruitment procedure is outlined in Figure 1.

#### Sample size

A total of 900 participants will be initially recruited to ensure the sample size is large enough to meet the study objectives.

Objective 3: the sample size required is given by the rule of thumb: *n* = 104 + k, where k is the number of predictors in the model.^18^ Therefore, with 20 predictors in the model, a sample size of 124 (CYP who have had COVID-19) would be required, not accounting for attrition.

Objective 5: the sample size required is given by *n* = 50 + 8 k, where k is the number of predictors in the model.^18^ Therefore, with 20 predictors, a sample size of 210 (CYP with or without a history of having COVID-19) would be required, not accounting for attrition.

#### Data collection

Cohort study data will be collected through a series of online, self-reported questionnaires. All surveys will be delivered through the Keele Health Survey data capture platform. Following a baseline questionnaire, participants will be sent a URL link by email to complete follow-up questionnaires at 3 months, 6 months, 9 months, and 12 months (see Table 1). Participants who do not complete the questionnaire will be sent a reminder email after 2 weeks, with no further follow-up.

**Table 1.** Data collection schedule (questionnaires)

Description	Measure	Baseline	3 months	6 months	9 months	12 months
**Primary outcome measure**						
Health-related quality of life	KIDSCREEN-10 Index (10 items, 0–5 scale)	✓	✓	✓	✓	✓
**Secondary outcomes measures**						
Symptoms	Long-COVID-19 symptom inventory (ISARIC/NICE)	✓	✓	✓	✓	✓
Health and social care use		✓	✓	✓	✓	✓
New diagnoses		✓	✓	✓	✓	✓
School absence and attainment		✓	✓	✓	✓	✓
**Baseline measures**						
Age		✓				
Sex		✓				
Ethnic group		✓				
Postcode		✓				
Height		✓				
Weight		✓				
Living status		✓				
Vaccination status (if applicable)		✓	✓	✓	✓	✓
If had COVID-19: type of acute symptoms; symptom duration; symptom severity; place of care (home, ward, ICU)^a^		✓	✓	✓	✓	✓
Pre-existing health conditions and prescribed medication	Inventory or medication list	✓				
Pre-existing mental health needs	Inventory or service use	✓				
Exposure to long-COVID in adults	Have any adults in your household had ‘long-COVID’? Yes, no, don’t know	✓				
Physical activity		✓				

^a^If participant reports new case of COVID-19 over the course of the study, this question will be asked again.

ICU = intensive care unit. ISARIC = International Severe Acute Respiratory and emerging Infection Consortium. NICE = National Institute for Health and Care Excellence.

All participants will be asked to complete each questionnaire regardless of their COVID-19 status. Additional questions will ask those who have had COVID-19 about nature, severity, duration of symptoms, and treatment. Participants will be grouped into COVID and not COVID groups, according to self-reported positive SARS-CoV-2 polymerase chain reaction (PCR) or lateral flow tests. If a participant reports a case of COVID in follow-up, they will be moved into COVID group for the remainder of the study.

#### Questionnaires

The questionnaires include the following measures: KIDSCREEN-10 Index^19,20^ (validated for ages 8–18 years), which measures HRQoL; an inventory of long-COVID symptoms based on the International Severe Acute Respiratory and emerging Infection Consortium (ISARIC) Covid-19 Paediatric follow-up protocol ^21^ and National Institute for Health and Care Excellence (NICE) COVID rapid evidence review;^22^ and questions about service utilisation, new medical conditions diagnosed since COVID, and school absence and attainment. Further baseline information includes the following: sociodemographic variables; vaccination status; characteristics of COVID; comorbidities and lifestyle; and psychosocial and behavioural factors. Participants will be asked to self-report if they have had a positive COVID-19 test result.

### Statistical analyses

Symptom information collected at each stage will be descriptively summarised for the entire sample, and the COVID-19 and non-COVID-19 groups. Rates of resolution of symptoms will be provided at each follow-up time point and compared between COVID-19 and non-COVID-19 groups.

Among the whole cohort, and by COVID-19 group, the incidence rates, and corresponding 95% confidence intervals, of symptoms at 4 weeks and 12 weeks after acute COVID-19 will be calculated. Incidence rates of long-COVID symptoms (any listed in the most recent NICE rapid evidence review^22^ and/or ISARIC questionnaire^21^ after at least 4 weeks after acute COVID-19) will be calculated at each follow-up time point among the COVID-19 group.

Among the COVID-19 group, logistic regression models will be used to obtain estimates of associations between candidate predictors and development of long-COVID at each of the follow-up time points, quantified in terms of odds ratios and corresponding 95% confidence intervals. Unadjusted associations will be obtained, followed by a full multivariable model. Decision on candidate predictors to be included in the full multivariable model will be driven by evidence from previous research, and clinical expertise.

To describe the physical, psychological, and social outcomes of long-COVID, results of health and healthcare utilisation at baseline and each follow-up point will be described for participants identified with long-COVID, and compared with those without long-COVID. Results will be presented descriptively and appropriate comparison methods used to compare between group differences.

Linear regression models will be used to obtain unadjusted associations (quantified in terms of regression coefficients and corresponding 95% confidence intervals) between candidate predictors and poor outcome in terms of HRQoL, as described by the KIDSCREEN-10 Index at 6 months' post-baseline among the entire cohort. Adjusted associations will then be obtained via a full multivariable model. Vaccination status and long-COVID will be entered into the model. The results of objective 3, clinical expertise, and PPIE input will decide which candidate predictors will be included in the full multivariable model. A further analysis will be conducted at 12 months' follow-up.

Primary analyses for objectives 3, 4, and 5 will be based on participants with complete data. Multiple imputation by chained equations, using 50–100 imputations, will be used to impute missing data, and analyses re-performed.

### Qualitative study

The qualitative study comprises semi-structured interviews, diary entries, and focus groups, and will run simultaneously with the cohort study.

#### Semi-structured interviews and diary entries

Cohort study participants who reported a positive COVID-19 test and symptoms lasting longer than 4 weeks will be invited to an interview. Approximately 20 CYP will be recruited using purposive sampling to ensure a range of characteristics; for example, age, sex, sociodemographic background, and symptom duration. Interviews will explore participants’ experiences of long-COVID and the impact it has had on their lives. Participants aged ≤15 years will be required to have an adult family member present, but CYP aged ≥16 years can choose to be interviewed accompanied or alone. If an adult is present and contributes to the interview, their perspectives will be included in the research data. Interviews will be digitally recorded, and the recordings will be transcribed and anonymised for analysis.

CYP who report experiencing symptoms or effects of long-COVID will be offered the opportunity to take part in a diary study to document their day-to-day experiences. Participants will be sent a workbook-style diary, which includes instructions, prompted pages and blank pages, and will be encouraged to write or creatively express their thoughts and experiences of long-COVID over a 2-week period. Completed diaries will be returned to the researcher and their contents will be analysed. These data will provide ‘real-time’ understanding of daily experiences of managing the impact of long-COVID and will supplement the interview data, supporting a richer understanding of participants’ experiences.

#### Focus groups

In addition to the interviews and diaries, 1–2 focus groups will be conducted with people who work with CYP in professional or third sector and voluntary roles. Approximately 12 participants will be recruited via purposeful sampling through professional networks. Prospective participants will be sent an information sheet and consent form via email, and can return their consent form via email to be included.

The focus group will be hosted virtually and will be digitally recorded. The questions will explore the perceived impact of long-COVID on CYP from the perspective of professionals involved in their health, education, and social settings. The topic guide will be used flexibly, in line with an inductive approach. Following the session, the recordings will be transcribed and anonymised for analysis.

### Qualitative analyses

Qualitative data will be analysed thematically using the constant comparison method.^23^ The three datasets will first be analysed separately and then mapped onto each other, allowing for a direct comparison of views and experiences. The data collection and analysis will be carried out concurrently to support development of themes and collection will continue until inductive thematic saturation is reached, the point at which no new codes or themes are developed through data analysis.^24^ Further analysis will involve exploring identified themes in relation to relevant theories of illness experience; for instance, biographical disruption^25^ and biographical contingency.^26^

The themes will be discussed with collaborators and the PPIE and stakeholder groups to explore their insight and interpretation. The final themes will be used to develop a conceptual framework illustrating the views of CYP, parents, and professionals towards the experiences and management of long-COVID.

### Data security

Data management will be carried out according to the data management plan, in accordance with Keele University Health and Social Care Research (HSCR) Standard Operating Procedures (SOPs). The study data will be stored on Keele University storage services and protected by industry standard security tools. All confidentiality arrangements adhere to relevant data protection regulations and guidelines (General Data Protection Regulation [GDPR], Caldicott, General Medical Council [GMC], Medical Research Council [MRC] UK policy). All information collected during the course of the study will be kept strictly confidential. All data used for analysis will be pseudonymised and will be stored separately from personally identifiable data. Study documentation will be stored for a minimum of 10 years after the full research programme has completed, when it will be destroyed.

### Ethical considerations

Parents, guardians, and CYP will be fully informed about the study. They will be sent information sheets and consent forms and will have the opportunity to ask any questions before they give consent. Following NHS Health Research Authority^27^ recommendations, age-appropriate assent or consent will be requested: CYP aged ≥16 years will be asked to complete a consent form, while CYP <16 years will be offered the opportunity to assent, while their parents will be asked to give consent for their child to participate. CYP who turn 16 years during the study will be offered the opportunity to reconsent.

Participants may feel fatigued or distressed or may disclose information about being at risk. The researchers are unable to give medical advice and will make this clear in all participant information sheets. Should participants become distressed during interviews, they will be given the opportunity to temporarily pause or end the interview. Participant documentation will include information about sources of support and signposts for further medical advice and, if necessary, the researcher will signpost the CYP and parent or carer to organisations that can provide support (for example, Kooth, Childline, NSPCC). Child safeguarding procedures are in place.

### Dissemination

SPLaT-19 findings will be presented at conferences and published in peer reviewed journals. The project team will work with the PPIE group to develop dissemination materials for lay audiences and clinical or professional groups; for example, social media posts, information materials, or other graphic materials.

## Discussion

SPLaT-19 will contribute further knowledge about the longer-term effects of an acute COVID-19 infection in CYP aged 8–17 years, including about the experiences of CYP with long-COVID and experiences of professionals involved in their care. While there are elements of the study that are similar to existing cohort studies, for example, the CLoCk study^5^ and research being carried out by Roessler and colleagues,^28^ SPLaT-19 also uses qualitative methods to explore the lived experiences of both CYP with long-COVID and professionals who care for them, which will also provide insight into the wider social impact of long-COVID for CYP. To the authors' knowledge, this has not been investigated elsewhere.

Recent developments in the UK policy regarding COVID-19, notably the CYP COVID-19 vaccination programme and the cessation of regular lateral flow testing across the nation, may affect recruitment and data collection for this study. Similarly, the recent spread of the less virulent variants of COVID-19 could also potentially affect the number of people who participate in the study. These are areas to consider as the study progresses. The generalisability of the study findings should also be considered. While the study will use sampling within the recruitment stages, particularly for the participants for interview, the recruitment area is limited to the West Midlands in England and, as such, the findings may not be representative of the whole population.

Following the widespread childhood vaccination programme and the recent waves of the Omicron variant, as the UK continues to live with COVID-19, it is crucial to understand how long-COVID affects people and what the best course of treatment is for those with the condition. The data collected from this study will promote a better understanding and definition of long-COVID, which will inform clinical practice and support services in developing beneficial treatment plans and interventions for future CYP living with long-COVID. These findings will also have much broader impact, being pertinent for the general public, including CYP with long-COVID and their families, policymakers, and for people in education and public health.

## Data

Any subsequent requests for access to the final study dataset from anyone outside of the research team (e.g. collaboration, joint publication, and data sharing requests from publishers) will follow the Keele CTU’s data sharing procedure.
